# Strong structuration analysis of patterns of adherence to hypertension medication

**DOI:** 10.1016/j.ssmqr.2022.100104

**Published:** 2022-12

**Authors:** Maureen Seguin, Jhaki Mendoza, Gideon Lasco, Lia M. Palileo-Villanueva, Benjamin Palafox, Alicia Renedo, Martin McKee, Dina Balabanova

**Affiliations:** a The Centre for Global Chronic Conditions, The London School of Hygiene and Tropical Medicine, UK; b College of Medicine, University of the Philippines Manila, Philippines; c Development Studies Program, Ateneo de Manila University, Philippines; d Department of Anthropology, University of the Philippines Diliman, Philippines; e Department of Social and Environmental Health Research, London School of Hygiene and Tropical Medicine, UK; f Department of Global Health & Development, London School of Hygiene and Tropical Medicine, UK

## Abstract

Achieving blood pressure control is among the highest priorities for reducing the burden of cardiovascular diseases globally. Control is poor in the Philippines, especially in socioeconomically marginalised communities. This paper explores long-term adherence to anti-hypertensive medication in these communities, identifying 4 distinct medication adherence patterns. We draw on Strong Structuration Theory to explore motivations of action for those who are consistently adherent, consistently non-adherent, and those who became more or less adherent over time. We employ longitudinal qualitative methods comprising repeat interviews and digital diaries collected over 12 months by 34 participants. Twelve participants were consistently adherent, 9 consistently non-adherent, 9 increasingly adherent, and 4 increasingly non-adherent. For the consistently adherent, positive views about prescribed medication and family support encouraged adherence. Conversely, negative views of medication and lack of family support were notable amongst the consistently non-adherent, along with resistance to accepting a ‘sick’ label. A shift toward positive views of medication was detected amongst those whose adherence improved, along with worsening health and increased family support. A decrease in financial resources drove some participants to become less adherent, especially if they already held negative views toward medication. This study sheds light on the variety of medication adherence patterns among poor people with hypertension in the Philippines, as well as the complex web of elements influencing their treatment choices. The results point to the potential for measures that address concerns about medicines and increase family support.

## 1 Introduction

Hypertension (HT) control plays a crucial role in reducing the burden of cardiovascular diseases (CVDs) globally (GBD 2016 Risk Factors Collaborators, 2017). As in many low- or middle-income countries (LMICs) control is poor in the Philippines (Palafox et al., 2016). Yet there is relatively little research from LMICs contributing to the global evidence base on how to improve it (Khatib et al., 2014; Maimaris et al., 2013). Two global systematic reviews (Khatib et al., 2014; Maimaris et al., 2013) on factors associated with HT awareness, treatment, and control identified only 20 studies conducted on populations in LMICs.

There is a lack of research on adherence to HT medication among poor populations in LMICs, though socioeconomically marginalised hypertensive individuals have particularly poor control of HT compared to their wealthier counterparts (Palafox et al., 2016). Nonetheless, it is possible to achieve good control in LMICs. A recent review of patient pathways for CVDs in Malaysia and the Philippines identified some examples of effective long-term management and control (Seguin et al., 2021). To achieve control, it is necessary to understand the diverse pathways that patients follow, particularly regarding adherence to HT medication. Previous work in the Philippines reveals how complex this is, reflecting patient conceptualisations of ‘high blood’ (Lasco et al., 2020) and their unique therapeutic itineraries (Mendoza et al., 2021).

Patient-level barriers to medication adherence include poverty and the resulting inability to pay for medication (Ambaw et al., 2012; Thomas et al., 2011), negative patient attitudes toward medication (Thomas et al., 2011), patient actions/inactions (‘drug holidays’/forgetfulness (Thomas et al., 2011)) and negative experiences with health services (Ambaw et al., 2012; Hassan et al., 2006; Thomas et al., 2011). Barriers at the health system level include incorrect implementation of clinical guidelines (Wang, 2003), geographic barriers (Ambaw et al., 2012) and complex medication regimes (Hassan et al., 2006). Conversely, adherence facilitators include support from family/friends (Osamor & Owumi, 2011), access to private sector healthcare (Dennison et al., 2007) and free medication (Yu et al., 2013).

Though these findings contribute toward our understanding of individual and health system barriers, it is often unclear how these operate in a given setting, and how individual-level characteristics are both shaped by, and impact upon, the social context framing the individual. For instance, although a link between patient attitudes toward medication and medication (non)adherence has been identified, less is known about how these attitudes are formed. Moreover, studies on hypertension medication adherence tend to be cross sectional, imposing a static view of medication adherence. There is comparatively little research on whether adherence (and elements shaping adherence) change over time.

The current evidence base on HT control in LMICs is largely atheoretical. A recent review on frameworks conceptualising health pathways of individuals diagnosed with CVDs (Seguin et al., 2020) identified only 15 studies, with the majority drawing on the common-sense self-regulation model (Leventhal et al., 2016), transtheoretical model (Prochaska & DiClemente, 1983) or theory of planned behaviour (Ajzen, 1991). As argued elsewhere (Seguin et al., 2020), these models largely conceptualise treatment pathways as driven by individual patient attributes such as attitudes and belief systems, with little appreciation of how these attributes are formed. Moreover, there is a lack of focus on the social, political and economic forces shaping CVD patient pathways.

This paper draws upon Strong Structuration Theory (Stones, 2005) to extend understanding beyond individual-level explanations of patient behaviour and recognize the non-linear nature of the HT medication adherence patterns. It explores the context shaping long-term medication adherence patterns of economically marginalised hypertensive individuals in the Philippines.

### 1.1 Theoretical background

The exploration and interpretation of drivers shaping medication adherence is facilitated by Strong Structuration Theory (SST) (Stones, 2005), based on Giddens' ‘Structuration Theory’ (Giddens, 1984). Central to Structuration Theory are the concepts of agency and structure; agency conceptualized as human action (and also individual knowledge about the rules which govern action), and structure as rules (procedures which perpetuate social life, including social conventions and official regulations) and resources (sources of power) (Giddens, 1984). Conceptualising society entirely from either the standpoints of agency or structure is problematic; the former ignores barriers which limit human agency while the latter affords no power to the individual to act freely. Structuration Theory attempts to overcome the dichotomy between structure and agency by proposing a ‘duality of structure’: structures are created by, and shape, human agency (Giddens, 1984). Thus, structures and agency are interdependent, mutually reinforcing, and have the capacity to evolve over time.

**Fig. 1:**
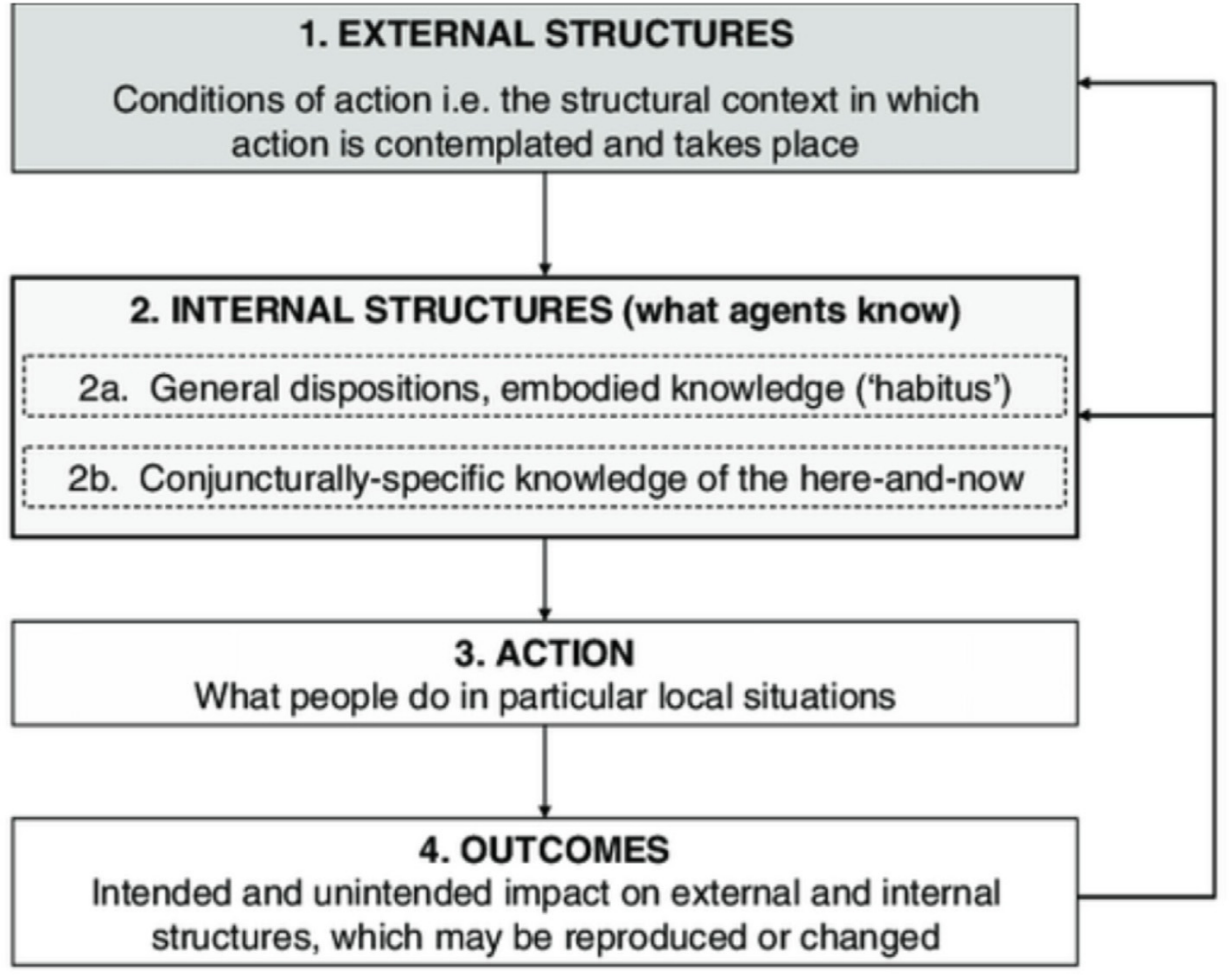
Elements of Strong Structuration Theory From Stones (2005).

Critics of Structuration Theory have pointed out that Giddens' concepts of structure and agency are difficult to operationalise, precluding empirical use (Archer, 1982; Stones, 2005). To address this, Stones (2005) amended Structuration Theory to make key concepts amenable to empirical research. Termed Strong Structuration Theory (SST), the theory consists of a ‘quadripartite cycle of structuration'consisting of 4 elements: external structures, internal structures, action/active agency and outcomes (Stones, 2005) (see Fig. 1).

External structures are conditions of action. They include traditions, norms, moral codes and established ways of doing things which exist independently of individuals. Though these shape human behaviour, external structures are also modified by the choices made by individuals (Stones, 2005). External structures are internalised by individuals in the form of beliefs and attitudes toward external structures, which are gained through interaction with external structures. Internal structures are ‘what agents know' and consist of two subtypes: (i) general dispositions, which are attitudes, opinions, beliefs and (ii) conjuncturally-specific, which is knowledge specific to situations. ‘Conjunctures’ between these two types of internal structures informs subsequent action as individuals draw on their knowledge of the social world (conjuncturally-specific knowledge) and general dispositions to choose action in a given context. Action/active agency, the third element of SST, is exercised by individuals drawing on their internal structures to act. Such actions produce outcomes, which either preserve and reproduce or alter existing structures (Stones, 2005). The quadripartite cycle of structuration offers a vantage point to understand and interpret the non-linearity of patient pathways. It facilitates focus on both the broad environmental context and individual-level interactions framing individual action.

SST has been used to theorize changes in health system and health delivery, specifically on the introduction of technology (Greenhalgh et al., 2013, 2014, 2019; Greenhalgh & Stones, 2010; Hvidt et al., 2021; Jeffries et al., 2017; Porter et al., 2018; Shaw et al., 2018, 2020) and policy (Hughes et al., 2022; Rushforth & Greenhalgh, 2020) into health systems. A smaller cluster of SST-informed literature has focused on persons with health conditions (and their caregivers), specifically on childhood obesity (Chan et al., 2010) and chronic conditions (Hinder & Greenhalgh, 2012).

## 2 Methods

This study was conducted as part of the mixed-methods RESPOND study, which generated evidence on the barriers to effective hypertension management faced by poor households in Malaysia and the Philippines (Palafox et al., 2018). Seven *barangays* (the smallest Filipino administrative structure) in urban Metro Manila and 8 urban and 15 rural barangays Quezon province were purposively selected to facilitate access by researchers. All barangays had high proportions of households qualifying for government subsidies, indicating low socioeconomic status. Within selected households, we included in our sampling frame adults aged 35–70 years who either had a history of hypertension diagnosis or were identified as hypertensive during screening. From all hypertensive members in selected households, one was randomly selected to participate in the study, yielding a quantitative sample of 444 participants. These participants completed quantitative baseline and follow-up surveys. In the former, participants could opt to take part in the qualitative components of the study, providing their contact and demographic details. This formed a qualitative sampling frame from which 42 participants were purposively selected to cover a range of ages (between 35 and 70 years), sex (male and female) and location (rural and urban). Participants were contacted by interviewers and asked to confirm their willingness to participate in the qualitative study. Interviewers explained the study, provided an information sheet and answered questions about the study. Following this, written consent was provided by each participant. This paper draws on data from the qualitative study.

Qualitative participants completed baseline and follow-up interviews (where possible) spaced 12 months apart, and mobile-phone digital diaries in the interim. Interviews were conducted by [author initials] and [author initials] in Tagalog between October 2018 and November 2019. Interviewees were experienced qualitative researchers who inputted into interview topic guides. Baseline interviews focused on perceptions of hypertension and its treatment, as well as contextual features affecting these perceptions. Follow-up interviews focused on issues identified in the baseline interviews and diaries as important to participants regarding HT treatment.

We outline our diary data collection process elsewhere, including communication patterns, message types, barriers and facilitators of usage and levels of engagement by participants (Mendoza et al., 2021). Briefly, the digital diaries were intended to facilitate participants to share their day-to-day experiences of living with HT with local researchers, who responded at least weekly to comments (Mendoza et al., 2021). The diaries were supported by a platform developed by On Our Radar (On Our Radar, 2021), a secure web-based portal which allowed researchers to send individual or group prompts eliciting participant views on HT. Participants could also send messages unprompted by researchers. Participants were provided with an infographic which instructed them to use the diary to share their personal experiences and/or feelings about their hypertension, and as patients at health facilities.

Interview and digital diary data were transcribed in Tagalog, translated to English and the transcripts validated by bilingual authors to ensure fidelity. Transcriptions were exported into NVivo, where coding was completed.

### 2.1 Analytical approach

We used ‘methodological bracketing’ to carry out context and conduct analysis (Giddens, 1984; Stones, 2005). The former focuses on the terrain of external structures which provides the rules and resources (as well as possibilities and limitations) for action to take place. Highlighting this context allows one to distinguish the influences on action which are independent from individuals. We draw on existing empirical work and external structures identified by participants to outline this context.

This is followed by conduct analysis which draws on digital diary and interview data, with particular attention on participants' actions regarding (non)adherence and internal structures. Within the internal structures, moments of ‘conjuncture’ between general dispositions and conjuncturally-specific knowledge are identified. These conjunctures drive action; in this case, patterns of adherence/non-adherence to hypertension medication. The medication adherence patterns of participants are conceived as ‘cycles of structuration’ each with distinguishing structures (both internal and external), conjunctures, and actions outlined. Participants who reported taking their medication every day (or nearly every day) were classified as ‘consistently adherent’ and those who never, rarely or occasionally took their medication as ‘consistently non-adherent.’ Those who became more adherent during or before the study period were categorised as ‘increasingly adherent,’ and those who became less adherent during or before the study period as ‘increasingly non-adherent.’

To inform context and conduct analyses, we coded interview and digital diary data according to SST components: external and internal structures, action, outcomes and instances of conjuncture. Participant's reported actions related to their (non)adherence to HT medication were coded as ‘action.’ Individual beliefs, attitudes and perceptions of HT medication and alternative treatments motivating these actions were classified as internal structures. Features of the context shaping these internal structures beliefs were coded as external structures. Moments of conjuncture included interactions with family, health professionals and media content. Pseudonyms are used in quotes below. Within quotes, ‘I’ refers to interviewer and ‘P’ to participant.

## 3 Results

Though 42 qualitative participants were recruited, 8 were excluded due to lack of follow-up data or unclear adherence patterns from baseline to follow-up, leaving 34 participants included. Thirty-four baseline and 29 follow-up interviews were completed, along with 33 digital diaries.

Characteristics of participants appear in the appendix. Participants ranged in age from 39 to 69 yeas, with an average age of 54 years. There were 21 women and 13 men. Seventeen participants each resided in rural and urban locations. Just over a third were consistently adherent to prescribed medication from baseline to follow-up, while 9 were consistently non-adherent. Another 9 were increasingly adherent, and 4 became less adherent. There were no obvious trends related to sex and adherence patterns. Those who became less adherent were younger than the average age of all participants. There was a trend of rural participants becoming less adherent or being consistently non-adherent, and of urban participants being consistently adherent. The small sample size precluded formal statistical tests of these trends.

### 3.1 Context analysis

#### 3.1.1 Media landscape

The interviews and diaries were replete with references to the media landscape as a source for health advice, with specific mentions of Facebook, YouTube, radio and television. Facebook messenger is available to use without data allowance for most Filipino smartphone users (Consunji, 2013), which has contributed to a proliferation of Facebook users: an estimated 88 million Facebook users are in the Philippines (Dean, 2022).

Specific social media personalities were named as influential in informing HT treatment decisions. Prominent personalities included celebrity doctor Willie Ong, who has a large presence on YouTube, Facebook, and until 2019 appeared on the popular ‘Salamat Dok’ (Thanks Doc) medical television program where he has promoted both ‘Western’ and natural remedies. Other personalities include Dr. Edinell Calvario, host of the ‘Healing Galing’ television program and YouTube channel, where she promoted herbal remedies and a product line. Health programs on the radio were also influential. ‘Radio Veritas,’ a Catholic radio station, was specifically mentioned as a source for herbal treatment advice.

#### 3.1.2 Deprivation and universal health coverage

The Philippines has one of the highest poverty rates in the Association of Southeast Asian Nations (ASEAN) region (World Population Review, 2022). During data collection, incidence of poverty (defined as insufficient income to meet basic food/non-food needs) among Filipinos was 21.1%, while the subsistence incidence (defined as insufficient income to meet basic food needs) was an estimated 8.5% (Republic of the Philippines Statistics Authority, 2021). By early 2021, these incidences increased to 23.7% and 9.9% respectively. The ‘Pantawid Pamilyang Pilipino Program’ (4Ps) Act, a mean-tested cash transfer program for low-income households has been in place since 2007 to address poverty. Among other conditions, the program requires recipients to attend monthly sessions which may include blood pressure readings.

The pervasively high levels of poverty paired with the historic lack of health insurance has led to high rates of out-of-pocket health expenditure in the Philippines (Department of Health, 2018). Such payments are catastrophic for Filipinos in poverty, who either do not to treat their illness or attempt to raise funds from their social network, appealing as a last resort to the PhilHealth national insurance program (widely viewed as insufficient to cover needs) (Lasco et al., 2021).

The 2012 Philippine Package of Essential NCD interventions (PhilPEN) program includes HT medication as an essential medicine to be provided free of charge at public sector health care facilities (Republic of the Philippines Department of Health, 2012). Universal Health Care was introduced in 2019, which was intended to expand access to services, protect against financial risk, and shift resources to primary care (Dayrit et al., 2018; Republic of the Philippines Department of Health, no date). Despite these policy developments aimed at removing financial barriers to HT medication, access remains limited as clinics frequently run short and either ration or pause distribution.

Taken together, the context framing participant action is characterized by pervasive deprivation, inadequate medication access, a lack of government support to meet health costs, and frequent exposure to a media environment presenting conflicting health advice. The cycles of structuration below trace how these features of the ‘strategic terrain of action’ inform medication choices taken by participants.

### 3.2 Conduct analysis

#### 3.2.1 Consistently adherent participants

Twelve participants were consistently adherent to their prescribed medication during the study. Interactions between participants and family reinforced participants' positive attitudes towards HT medication, widely referred to as ‘maintenance.’ Such moments of conjuncture were omnipresent in the data, with many participants mentioning family members as helping them to stay ‘on track’ in a variety of ways, including giving medication reminders, paying for medication, and/or providing transportation to appointments. Rodrigo explained that his adherence was facilitated by his wife's work at the health centre: “my maintenance is still ongoing because my wife works here and this is where we get my medicine.” Participants also referenced previous interactions with ill family members as informing their current behaviour. For instance, Fernandez mentioned his father's stroke as motivation for controlling his HT:

I'm worried because my father had a stroke before, and we took care of him for five years. He was in a bed-ridden situation. […] I’m worried that might happen to me also, and my children have to take care of me. […] [H]aving a stroke is scary, especially when I saw what my father had to go through. He had a hard time, and we had a hard time taking care of him.

The consistently adherent group held positive perceptions of prescribed medication, viewing it as an effective measure to decrease elevated BP readings. For instance, Joyce remarked, “when I take it the result [BP measurement] really goes down.”

Many consistently adherent participants reported seeking health material on Facebook, YouTube, radio and television, constituting multiple moments of conjuncture which fed into medication choices. Health content supported positive impressions of both herbal remedies and prescribed medication. For instance, Christina supplemented her daily prescribed medication with a ‘healing oil’ purchased from the Healing Galing line of products when she felt symptomatic. Others used the herbal supplement ‘MX3,’ citing adverts as motivating use. Hazel explained that she tried ‘MX3’ (a herbal supplement) after hearing adverts for it on the radio: “[I]: Why did you try [‘MX3’]? [P]: Just to see if it will work. Because I hear it in the radio that it’s good for high blood and for many other illnesses.” In her digital diary, Nicole mentioned that she read on social media the benefits of lemon and honey, as well as green or black tea in treating HT, and had subsequently modified her own diet and that of her husband. A few participants specifically mentioned celebrity doctor Willie Ong as a source of information on the treatment of health conditions with herbal remedies and diet, revealing the reach and influence of such celebrity doctors on patient choices.

Though the consistently-adherent patients held positive attitudes towards prescribed medication and received support from family members to adhere, future adherence was uncertain. The fluctuating and often insufficient supply of free HT medication at clinics threatened adherence. Participant Gloria, a barangay health worker based at a health facility, shared that clinics often ran short, forcing staff to distribute as few as 5 tablets per patient:

If we are really low on medicine, sometimes we just give 5 tablets, since the maintenance is just once a day. […] When they consume their medicine, we tell them to come back after, so that we can give medicine to everyone.

This scenario required patients to travel to clinics to receive free medication as frequently as every 5 days, which was infeasible for some. It pushed many participants to purchase medication that was supposed to be provided free of charge, presenting a financial burden difficult to manage. This was the case with Nicole, who often had to purchase her prescription, at 6 pesos (∼0.12 USD) per tablet, as well as her husband's medication. She explained, “We just buy outside, rather than dying if you can't take [the medication]. […] [T]hose who are poor, who can't keep buying their maintenance, their hypertension becomes worse and worse.” Some adherent participants, such as Alejandro, bought *all* their HT medication out-of-pocket. Alejandro spent approximately 400 Philippine pesos (∼8 USD) per month. When asked about the financial impact, he stated, “it hurts a little but what can I do?”

Financial challenges were particularly acute for multi-morbid participants, who took several daily medications which were expensive to buy. Nicole explained in her digital diary,

I've had many things tested, the fatty liver is the one being treated right now. […]. The medicine is so expensive, 58.00 [Philippine pesos, ∼1 USD] plus the Vitamin E 5.50 [Philippine pesos, ∼0.10 USD] as well. Plus the Losartan [prescribed medication for HT] 9.00 [Philippine pesos, ∼0.18 USD] a day.

Despite the lack of consistently available medications, even the sporadic provision of free medication was recognised by participants as facilitating their adherence. Without free medications, Crisanto speculated that he would not treat his HT at all: “I don't have any means to buy medicines.”

#### 3.2.2 Consistently non-adherent participants

Nine participants were non- or partially-adherent to prescribed HT medication during the study. They either dismissed the disease altogether (thus not treating it through any method) or consumed a variety of certain foods and herbal remedies perceived to control HT either in their natural form or in branded capsules containing herbs. Those who consumed these supplements did so in variable degrees of regularity. Like the consistently adherent participants, the media shaped perceptions of HT treatment. Non-adherent participants cited social media sources (frequently YouTube and Facebook) as a source of information about herbal approaches. For instance, Cecilia explained that she does “research of herbal medicine on Facebook.” She elaborated,

We boil ginger. Salabat and ginger then lemon grass. When I cook food, lemon grass is always included. I have lemon grass even in vegetable and fish. [I] What is it for? [P] High blood. According to herbal doctors. […] [I] Who was the doctor online? […] [P] Willie Ong.

The choice to not take prescribed medication regularly or ever during the study stemmed from two internal structures: participants’ self-perceptions as healthy individuals and negative perceptions of prescribed medication. Negative perceptions of prescribed medication (and conversely, positive impressions of alternative treatments) were largely informed by the media (notably Facebook and social media personalities). Interactions with family members tended to reinforce negative attitudes toward medication. These points are elaborated upon below.

Several consistently non-adherent participants linked their opposition to taking medication to their self-perception as a healthy person. As they did not identify as sick, participants reasoned that there was no need to take medication daily. They equated taking medication with accepting the label of a sick person; of reifying HT as an illness which they had. Denial that HT was a serious concern, and/or that they were truly hypertensive often appeared alongside sentiments equating medication consumption with illness.

These sentiments are exemplified in Angela's interviews. In her baseline interview Angela shared her perception that taking a daily medication indicated that one had a diagnosed disease. As she chose not to take her prescribed medication, even after hospitalization for dangerously high blood pressure, she rationalised that she did not have a formal HT diagnosis. During her follow-up interview, she elaborated on this line of thinking:

I do not consider [HT] as a disease. […] I know that you have a disease when you have to take medicine every day because you are afraid that it will be worse when you don’t keep track of it. But I don’t have one. Look at me, ever since I've been interviewed last year, I haven’t taken any medicine for high blood. I don’t buy the medicine that they prescribed me from Dalandanan Hospital.

The idea that taking medication contributed to illness was evident in this group. It was rationalised that focusing on HT and taking medications caused stress, which then exacerbated the HT. As such, *ignoring* medication was paradoxically viewed as a way of treating HT. For instance, Cecilia chose to avoid medications and thinking about her HT diagnosis due to her belief that doing so would increase her stress level, thereby elevating her blood pressure readings. In her baseline interview she reasoned, “I ignore my medicines [for] high blood. I just did it because if I will think about it, I will be more high blood. […] If you take maintenance, your high blood will not leave you.“Her views at follow-up showed her reluctance to take medication was linked to her reluctance to reify her diagnosis. When asked whether her physician supported her decision to take medication only sporadically, she responded: “It's my decision. My doctor said that I should take maintenance, but I don't want to depend on it. […] *I am not used to the feeling that I take medications*” (italics added). This passage shows that Cecilia's opposition to taking medication stems partly from her discomfort at seeing herself as someone who takes medications. It also shows a perception that taking medication leads to dependence, as elaborated further below.

Cecilia's attitude toward HT medication was reinforced through interactions with her husband, also hypertensive. Cecilia explained that her husband's HT was quite serious, and he took daily medication over a prolonged period of time. When he experienced some liver problems, Cecilia and her husband attributed this to his history of taking HT medication. She stated, “Because look at what happened to my husband. Because of taking maintenance, his liver become affected. […] That's why I don't like to take much maintenance.” Cecilia's understanding of her husband's reaction to maintenance, paired with her own negative attitude toward medication described above, informed her decision to take HT medication only sporadically. Cecilia mentioned occasions where she wished to take maintenance due to perceived symptoms and being unable to access it (possibly due to medication shortages described in the context section). On such occasions, Cecilia took her husband's medication.

Cecilia's impression that long-term adherence was harmful to organs was common amongst the consistently non-adherent group. The synthetic nature of medication was viewed as causing this damage, and as a reason to distrust it. For instance, when Daniel was asked about his thoughts on HT medications, he stated:

It’s synthetic. It would have been good if it’s herbal. I wouldn’t mind if I have to [consume] it regularly. But if it’s synthetic like made in laboratories I don’t like the idea of taking it long term. […] It can also damage our vital organs. […] It can damage the kidneys things like that, so I thought to myself, “why should I take medications given those things?”

Participants specifically mentioned concerns that HT medication would damage their kidneys and livers, taken long-term.

In contrast, herbal remedies and the consumption of certain foods to treat hypertension were viewed as posing no risk to internal organs. Moreover, this course of action seemed to present much less risk to participants' self-perceptions as healthy individuals. The interviews were replete with statements on the health benefits of certain foods and herbal remedies, though there was a lack of clarity over what the remedies treated specifically. For instance, Daniel explained that he ate mangosteen and drank tea made with the rind for the health benefits. When the interviewer asked what specifically this treated he responded, “Not really sure what it is for. It's for whatever you want it to be.” He nonetheless believed that the treatment was effective, as did other participants using herbal remedies.

Non-adherent participants also perceived that taking medication long-term made one dependent on it. Antonio's recollections about his HT diagnosis approximately 1 year before the baseline interview exemplify this. Having received a prescription, he consumed the first prescription course but then declined to renew his prescription due to concerns over reliance on the drug. He stated at baseline, “I just don't want my body to get used to taking medicine.” His attitude was unchanged at the follow-up interview, where he said “I don't like to take my medicine. I've been given some maintenance, but I tend not to take it because I don't want my body to depend on medications.”

In a sharp contrast with adherent participants, experiences with family members who became ill or died from CVDs did not appear to influence the behaviour of non-adherent participants. For instance, at the time of the baseline interview, Angela was caring for her mother who had suffered a stroke. Her mother died months later, which Angela believed caused her own blood pressure to increase to a point where she was hospitalised. Nonetheless, neither her mother's condition nor her own health emergency led Angela to become more adherent; at follow-up she was still only taking medication when her blood pressure readings were high.

#### 3.2.3 Increasingly adherent participants

Nine participants reported becoming more adherent over the course of the study, though some had not reached full adherence. At baseline, many of these participants were taking prescribed medication only when their blood pressure readings were high, forgot to take it for days at a time, or took regular ‘drug holidays’ to give their organs a ‘break’ from medication. Like the consistently non-adherent, many reported taking alternative treatments including MX3, infusions of Guava leaves, lemongrass, serpentina and other herbal remedies, and/or calamansi juice. Others reported controlling their HT through diet modification or rest. At follow up, most of these participants reported taking their prescribed medications every day, or at least were more adherent than at baseline. Almost all continued to take alternative therapies. Key structures and conjunctures which appeared to influence this change are described below.

Improvements in adherence paralleled shifts in participant perceptions on medication. At baseline, these participants had ambivalent attitudes toward prescribed medication for HT. Though some referenced the benefits of medicine, these were tempered by sentiments of being ‘fed up’ with medication and concern over organ damage. At follow-up, participants recognised that medication decreased blood pressure readings and seemed less concerned with side effects. Many participants continued to supplement their medication regimes with herbal remedies and following a healthy diet.

This change in attitude appeared to stem from increased trust in health professionals, gained through long-term interaction with particular health facility staff. This is exemplified in James's case, whose attitude toward his HT medication shifted greatly between the two time points. At baseline, he voiced concern over potential side effects, and stated his intention to not take his medication regularly. He shared, “the side effect is when your internal organs are damaged. […] Because I am really afraid of that. You already have a disease then you will take a medicine that will destroy [internal organs].” Moreover, James was concerned over the expense of the medication. Despite these barriers, James had a physician he went to regularly and had done for some time. After the baseline interview, his doctor had convinced him that long-term HT medicine was not harmful to his internal organs, and any expense incurred to obtain medication paled in comparison to the costs of treating a stroke. James was persuaded by these arguments and was much more adherent at follow-up. In his follow-up interview he explained,

[P] That Dr said it is better to spend a little on medicine than to spend more once you had a stroke. That's why I push myself to take medicine since there are no side effects in our body. [I]: It's Doctor [name of doctor], right? [P]: Yes, [name of doctor] is my doctor. I always to go him/her when I feel something because he/she really knows about my body. [I]: Yes, he/she knows about your history. [P]: Yes, the history.

This passage shows that patients’ dispositions toward medication can be changed in interactions with health professionals; new information may change their understanding of their condition and thus behaviour.

Besides interaction with health professionals, the other driver for increased adherence was family interactions. Increased family support for adherence for this group typically followed a health emergency which underscored the importance of taking medication. For instance, Theresa had been taking her HT medication approximately every second day until she had an episode of dangerously high HT. Her child, who was present during the episode, got her medical help. After she was successfully treated, all of Theresa's adult children insisted she take her medication daily and see a physician regularly to monitor her medication. They facilitated the latter by providing transportation and accommodation in the city of Lucena, even though this was a considerable distance from Theresa's rural home in the Municipality of Samapaloc. Theresa's children chose for her to see a physician in Lucena because one of her children worked there as a nurse, who also covered her medical expenses. Theresa's grandchild, also a nurse, also reinforced the importance of taking medication. Theresa reflected on her family's influence over her medication adherence:

I believe in them since there are two mouths bothering me, my child and my grandchild. […] I believe in them since they’ve studied about it even if they are not doctors. They are in the field of medicine, that’s why they keep track of my condition.

Without this support from her family (notably those employed in the health system), Theresa speculated that she would be unlikely to take her medication regularly because she normally felt fine. This case demonstrates the importance of family in shifting patient behaviour.

#### 3.2.4 Increasingly non-adherent participants

Four participants became less adherent during the study. At baseline, these participants took prescribed medications frequently or almost always. They also took herbal remedies, consumed teas and juices, vinegar and garlic to treat their HT, and had modified their diets. At follow up, consumption of herbal remedies and diet modifications were unchanged. However, the participants were less adherent to their prescribed medications than at baseline, with some participants ceasing these treatments entirely.

At both baseline and follow up, participants in this category held negative impressions of prescribed medications. They felt ‘fed up’ with taking daily medications and shared many of the same perceptions of medication as the consistently non-adherent participants (taking medication led to dependence and organ damage). For instance, though she accepted her medication was effective in reducing her blood pressure, Camille stated at baseline,

When I remember that I have to drink it every day, I just become more fed up. It’s been such a long time. Like I said before, what is the medication that I can take to make high blood totally disappear so I won’t have to take another medicine?

The one exception to these sentiments was Bernard, who at baseline had a positive view of medication. He viewed his Losartan as effective in treating his HT symptoms without any side effects and intended to stay on the medication long-term. After the baseline interview, Bernard shared in his digital diary that he was experiencing a throat issue, which he attributed to his HT medication. He messaged, “It has been a while that I have been taking my maintenance that there seems to be a side effect on me, so I plan to stop taking Losartan and I plan to make lemon juice.” The family were unable to afford for Bernard to see a doctor about the throat issue. At follow-up, Bernard had a very negative view of prescribed HT medication due to this perceived side effect and had thus stopped taking it.

Bernard's lack of financial resources for health care was common amongst the increasingly non-adherent group. Their financial situation seemed to worsen between baseline and follow up, though financial stressors were also apparent at baseline for this group. Participants in all adherence groups frequently mentioned that clinics ran out of the medication which was supposed to be provided free of charge. Although other participants were able to pay for medication occasionally when clinics ran out (or rely on family members to pay), the increasingly non-adherent group could not do so at follow-up. Camille, who occasionally worked as a cleaner and did intermittent ‘cash for work’ jobs in her barangay, was adherent at baseline. At that time, she explained that when her nearest health centre ran out of Losartan, she bought it at a drugstore instead: “It's free here in the health center. If I run out then I buy, it's only 50 pesos [∼1 USD] for 1 blister pack.” She explained that she sometimes bought only 2 pills at a time, for 10 pesos. At follow up, Camille could no longer afford to buy HT medication outright and had been advised by a health professional to drink pineapple juice or vinegar if she ran out of Losartan and there was no free medication available. She explained,

[S]ince the time that the center ran out of supplies, sometimes I haven’t been able to buy my maintenance because I don’t have enough money. There are times that I haven’t been able to take my maintenance for three days. If I have enough money, I buy two or four tablets. But for instance, if it is harvesting season, I don’t have enough money to buy my maintenance.

Increasing pressure to work to make up the financial shortfall also interfered with activities such as blood pressure monitoring and medication reviews at clinics, as participants prioritised work over their health. Nonetheless, participants recognised that taking medication could help rather than impede their work. For instance, when asked whether vinegar or Losartan was more effective in treating her HT Camille stated, “Losartan. It's better if I'm able to take it every day because I can function at my work well.”

## 4 Discussion

This paper draws upon Strong Structuration Theory to interpret the long-term patterns of medication adherence of economically marginalised hypertensive individuals in the Philippines. The quadripartite cycle of structuration offers a vantage point to understand and interpret the patient adherence pathways over time, conceptualising these pathways as shaped by internal and external structures. This theoretical position allowed us to delineate the complex web of elements enabling and disabling adherence along the 4 adherence patterns found. This approach represents an advance over previous frameworks to exploring CVD patient pathways which typically do not consider the wide range of interrelated forces which may shape these pathways and are often unsuited for assessing longitudinal changes in adherence patterns (Seguin et al., 2020).

### 4.1 External and internal structures

Four patterns of action were evident: participants who were consistently adherent, consistently non-adherent, increasingly adherent, and increasingly non-adherent. The adherent group held positive attitudes about HT medication, consistent with the wider literature on the link between perceptions of and adherence to CVD medication (Dhar et al., 2017; Leslie et al., 2019). This group also referenced the importance of family support in taking medications regularly, as evidenced elsewhere (Dhar et al., 2017; Schwalm et al., 2019).

Many of the same external and internal structures relevant to both the adherent and non-adherent groups. Whilst the former held positive attitudes toward prescribed medication, the latter held negative attitudes. Two distinct negative sentiments were evident: that long-term medication led to dependence, and medications were harmful because they were synthetic. The synthetic nature of prescribed medications was linked by non-adherent participants to organ damage, primarily kidney damage. This perception may relate to a generalized popular association in the Philippines between overmedication and kidney problems (Tan, 1999).

These negative views of medication amongst the non-adherent were reinforced by media sources, consistent with other findings on the impact of internet-based media on hypertension medication adherence (Weaver et al., 2009). A lack of support for adherence from family was notable among the non-adherent. Those in our sample who became more adherent specifically referenced worsening health, along with support from family and physicians as motivating adherence. This trio of features (worsening health, family support, and trust in health professionals) contributed to changing views of medication from negative to positive.

Throughout the data, the sporadic availability of free HT medication and financial burden of purchasing medication was a salient theme across all adherence groups. Even among the consistently adherent, this context was referenced as a barrier to future adherence. For the increasingly non-adherent participants, this external structure, paired with negative perceptions of HT medication discouraged adherence to the point that some discontinued their treatment. This finding is mirrored in other research; a review of systematic reviews of adherence to CVD medication showed a clear relationship between having to pay for medication and poor adherence (Leslie et al., 2019). Moreover, the multi-country Prospective Urban and Rural Epidemiology study found that a high proportion of patients with non-communicable diseases in LMICs reported not taking medication due to costs (Murphy et al., 2020). The overrepresentation of rural participants in the increasingly non-adherent and consistently non-adherent groups may indicate a particular lack of HT medication especially in rural areas.

The inconsistent availability of free medication contradicts the aims of the PhilPEN program (Republic of the Philippines Department of Health, 2012) and the UHC Bill, both intended to protect Filipinos from catastrophic health costs (Dayrit et al., 2018). Strengthening the implementation of these policies at the local level would support medication adherence and financial protection of hypertensive individuals in poorer communities. This has become more crucial as more Filipinos have been pushed into poverty because of the COVID-19 pandemic (Lema, 2021), which will only increase the financial barrier to accessing medication.

The importance of the media landscape as an external structure was particularly striking for the consistently adherent and consistently non-adherent groups. Both groups received and internalised positive messages about herbal remedies from well-known media personalities in the Philippines, through radio, TV, and social media. We observed that this external structure both encouraged and discouraged adherence, demonstrating Giddens’ view of external structures as both enabling and constraining (Giddens, 1984).

This paradoxical feature of external structures (as both enabling and constraining) is important to consider in reflecting on outcomes of participant action. Participants belonging to all adherence categories were engaged in a media landscape which both supported and discouraged the usage of prescribed medication, with plenty of content encouraging the usage of herbal remedies. Such messaging facilitated action in accordance with the content, for instance, by using herbal products solely or alongside prescribed medication. Other features of the context (widespread poverty and lack of effective policy solutions to enable access) tended to constrain participant adherence. Nonetheless, this did not mean that action was completely dictated by these external structures. Rather, our observation that some participants became more or less adherent over the course of the study indicates shifts in internal structures, spurred through social encounter with others. Increased support from health professionals and family seemed to encourage adherence amongst some of the study participants through a shift in their dispositions towards prescribed medication.

### 4.2 Theoretical reflections

The concept of external structures, defined as conditions for action, is nuanced and we recognize different interpretations are possible. Additional external structures bearing upon medication adherence besides the ones discussed here may play a role in explaining patient behaviour. A second challenge in applying SST was the difficulty in tracing the ‘origins’ of some internal structures. Participants who were consistently non-adherent largely saw themselves as healthy. However, we were unable to discern how this self-perception was formed, either through interaction with others or external structures. This self-perception may relate to the role of the unconscious in behaviour, recognised by Stones (2005) as an element which can play an autonomous role in action. Even with sufficient probing, it may not be possible for a participant to articulate the roots of perceptions. Though the digital diaries allowed researchers to frequently contact participants, as we report elsewhere (Mendoza et al., 2021) there was a lack of engagement by participants, precluding rich data on some rationales behind attitudes toward medication and self.

Despite these challenges, applying SST concepts illuminated the complex web of elements shaping medication adherence over time. It is well-suited for exploratory research into the choices people make, and the multiplicity of influences behind those choices.

### 4.3 Limitations

Some of the meanings in Tagalog may have been compromised via translation into English. A lack of follow up data and/or a lack of clear adherence pattern were observed for 8 of our original sample of 42, leading us to exclude them. This may have decreased the depth of our data. As more fully outlined elsewhere (Mendoza et al., 2021), engagement in our digital diaries was low, lessening our ability to collect detailed longitudinal data on adherence. As referenced above, it is possible that we may have missed key features of the context which nonetheless help explain HT medication adherence in our sample. As this is a relatively small qualitative study, the empirical findings cannot be generalized to other populations. However, the broad findings that a nuanced web of interconnected elements shape adherence behaviour amongst poor hypertensive residents in the Philippines may resonate in other low- and middle-income settings. A key strength of our work is the theoretical approach taken. Strong Structuration Theory has potential to be used elsewhere to illuminate the web of elements which drive patient choices in innumerable settings.

## 5 Conclusion

This paper explored drivers of long-term adherence patterns of economically marginalised hypertensive individuals in the Philippines. The variety identified both in patterns and drivers of patterns underscore the need for diverse interventions to improve or support adherence among hypertensive persons. We found that attitudes toward medication strongly influences adherence, yet these attitudes can change. Health care professionals involved in hypertension treatment should thus work to foster positive attitudes toward medication among patients, which would be bolstered by involving family members and (social) media. Moreover, structural change to ensure the availability and affordability of medicine (as set out in both PhilPEN and the principles of UHC to which the Philippines has agreed) would remove major barriers faced by socioeconomically marginalised groups, while supportive external influences, such as from family and media, could be harnessed to encourage individuals to increase and maintain adherence.

## Declaration of interests

LMP-V has been involved in clinical trials of antihypertensive medications that receive some funding from industry. No other authors declare conflicting interests.

## Author contributions

MS: Conceptualisation, Formal analysis, Writing – original draft preparation.

JM: Methodology, Data curation, Writing – Review & editing.

GL: Methodology, Data curation, Writing – Review & editing, Supervision.

LMPV: Conceptualisation, Writing – Review & editing, Supervision, Funding acquisition.

BP: Conceptualisation, Methodology, Writing – Review & editing, Supervision, Funding acquisition.

AR: Conceptualisation, Methodology, Writing – Review & editing, Supervision, Funding acquisition.

MM: Conceptualisation, Writing – Review & editing, Supervision, Funding acquisition.

DB: Conceptualisation, Writing – Review & editing, Supervision, Funding acquisition.

## Funding source

The authors disclose receipt of the following financial support for the research, authorship, and/or publication of this article: 10.13039/100010269Wellcome Trust/Newton Fund-MRC Humanities & Social Science Collaborative Award scheme [grant number 200346/Z/15/Z]. The funders played no role in the study design, in the collection, analysis and interpretation of data, in the writing of the articles, nor in the decision to submit it for publication.

## Declaration of interests

LMP-V has been involved in clinical trials of antihypertensive medications that receive some funding from industry. No other authors declare conflicting interests.

## Ethical statement

Our study was approved by the Observational Research Ethics Committee at the London School of Hygiene and Tropical Medicine and the Research Ethics Board at the University of the Philippines Manila.
