# Supplementary material for: Strong structuration analysis of patterns of adherence to hypertension medication
Source: SSM Qual Res Health. 2022 Dec;2:None. doi: 10.1016/j.ssmqr.2022.100104 (PMC9748305; doi:10.1016/j.ssmqr.2022.100104)
Supplement: Multimedia component 1 [file mmc1.docx]

**Appendix: Characteristics of participants**

| **Pseudonym** | **Age** | **Gender*** | **Barangay/ municipality (City/ Province)** | **Rural/ Urban** | **Occupation**** | **Diagnosis status** | **Adherence Pattern***** |
| --- | --- | --- | --- | --- | --- | --- | --- |
| Fernandez | 64 | M | Sampaloc (Quezon) | Rural | Rice farmer, buys and sells coconut | Unaware | CA |
| Eleanor | 47 | F | Sampaloc (Quezon) | Rural | Homemaker, farmer, cash for work scheme | Aware | CA |
| Christina | 52 | F | Sampaloc (Quezon) | Rural | Runs a store, takes in laundry | Aware | CA |
| Rodrigo | 53 | M | Dalandanan (Valenzuela) | Urban | School bus driver | Aware | CA |
| Alejandro | 66 | M | Lingunan (Valenzuela) | Urban | Unknown | Aware | CA |
| Jomel | 64 | M | Lingunan (Valenzuela) | Urban | Retired food factory worker | Aware | CA |
| Nicole | 58 | F | Parada (Valenzuela) | Urban | Former midwife | Unaware | CA |
| Joyce | 62 | F | Parada (Valenzuela) | Urban | BHW | Aware | CA |
| Gloria | 60 | F | Parada (Valenzuela) | Urban | Former BHW | Aware | CA |
| Beth | 45 | F | Parada (Valenzuela) | Urban | Unknown | Aware | CA |
| Crisanto | 55 | M | Punturin (Valenzuela) | Urban | Runs a 24-hour store | Aware | CA |
| Hazel | 44 | F | Punturin (Valenzuela) | Urban | BHW | Aware | CA |
| Jasmine | 42 | F | Perez (Quezon) | Rural | Barangay Councilor | Aware | CNA |
| Rosario | 56 | F | Perez (Quezon) | Rural | Unknown | Unaware | CNA |
| Ophelia | 61 | F | Perez (Quezon) | Rural | Unknown | Aware | CNA |
| Jose | 61 | M | Perez (Quezon) | Rural | Coconut and rice farmer, sells coal and bananas | Unaware | CNA |
| Antonio | 44 | M | Perez (Quezon) | Rural | Unknown | Aware | CNA |
| Maria | 62 | F | Perez (Quezon) | Rural | Vegetable/coconut farmer | Aware | CNA |
| Angela | 53 | F | Lingunan (Valenzuela) | Urban | Owns and operates sari-sari store, delivers water and gas, sells balot. | Unaware | CNA |
| Daniel | 61 | M | Parada (Valenzuela) | Urban | Former factory worker | Aware | CNA |
| Cecilia | 45 | F | Parada (Valenzuela) | Urban | Unknown | Aware | CNA |
| Bernard | 39 | M | Perez (Quezon) | Rural | Fisherman | Aware | LA |
| Camille | 50 | F | Sampaloc (Quezon) | Rural | Cleaner, farmer, cash for work scheme | Aware | LA |
| Jerome | 52 | M | Sampaloc (Quezon) | Rural | Unknown | Aware | LA |
| Katherine | 44 | F | Dalandanan (Valenzuela) | Urban | Chauffeur, takes in laundry | Aware | LA |
| Benilda | 51 | F | Perez (Quezon) | Rural | Farmer | Aware | MA |
| Patricia | 60 | F | Perez (Quezon) | Rural | Owns and operates sari-sari store, sells sweetened banana | Aware | MA |
| Andres | 64 | M | Perez (Quezon) | Rural | Retired artist | Aware | MA |
| James | 64 | M | Sampaloc (Quezon) | Rural | Produces coal | Aware | MA |
| Theresa | 69 | F | Sampaloc (Quezon) | Rural | Owns and operates sari-sari store, raises pigs, former farmer | Aware | MA |
| Imelda | 48 | F | Bignay (Valenzuela) | Urban | BHW, cook, sells balut and viands | Aware | MA |
| Ernesto | 50 | M | Dalandanan (Valenzuela) | Urban | Former driver at a public school | Aware | MA |
| Perlita | 49 | F | Dalandanan (Valenzuela) | Urban | Vendor, selling goods at home | Aware | MA |
| Jennifer | 48 | F | Punturin (Valenzuela) | Urban | Unknown | Aware | MA |
| *M = male, F = female | | |  |  |  |  |  |
| **BHW = Barangay health worker | | | |  |  |  |  |
| ***CA = consistently adherent, CNA = consistently non-adherent, LA = less adherent, MA = more adherent | | | | | | | |
